# Attempts at the Characterization of In-Cell Biophysical Processes Non-Invasively—Quantitative NMR Diffusometry of a Model Cellular System

**DOI:** 10.3390/cells9092124

**Published:** 2020-09-19

**Authors:** Weronika Mazur, Artur T. Krzyżak

**Affiliations:** 1 Faculty of Physics and Applied Computer Science, AGH University of Science and Technology, ul. Reymonta 19, 30-059 Cracow, Poland; 2 Faculty of Geology, Geophysics and Environmental Protection, AGH University of Science and Technology, al. Mickiewicza 30, 30-059 Cracow, Poland; akrzyzak@agh.edu.pl

**Keywords:** constant gradient, time-dependent diffusion coefficient, in-cell diffusion

## Abstract

In the literature, diffusion studies of cell systems are usually limited to two water pools that are associated with the extracellular space and the entire interior of the cell. Therefore, the time-dependent diffusion coefficient contains information about the geometry of these two water regions and the water exchange through their boundary. This approach is due to the fact that most of these studies use pulse techniques and relatively low gradients, which prevents the achievement of high *b*-values. As a consequence, it is not possible to register the signal coming from proton populations with a very low bulk or apparent self-diffusion coefficient, such as cell organelles. The purpose of this work was to obtain information on the geometry and dynamics of water at a level lower than the cell size, i.e., in cellular structures, using the time-dependent diffusion coefficient method. The model of the cell system was made of baker’s yeast (*Saccharomyces cerevisiae*) since that is commonly available and well-characterized. We measured characteristic fresh yeast properties with the application of a compact Nuclear Magnetic Resonance (NMR)-Magritek Mobile Universal Surface Explorer (MoUSE) device with a very high, constant gradient (~24 T/m), which enabled us to obtain a sufficient stimulated echo attenuation even for very short diffusion times (0.2–40 ms) and to apply very short diffusion encoding times. In this work, due to a very large diffusion weighting (*b*-values), splitting the signal into three components was possible, among which one was associated only with cellular structures. Time-dependent diffusion coefficient analysis allowed us to determine the self-diffusion coefficients of extracellular fluid, cytoplasm and cellular organelles, as well as compartment sizes. Cellular organelles contributing to each compartment were identified based on the random walk simulations and approximate volumes of water pools calculated using theoretical sizes or molar fractions. Information about different cell structures is contained in different compartments depending on the diffusion regime, which is inherent in studies applying extremely high gradients.

## 1. Introduction

Water in cells is very important for many processes, including cell division [1]. Water permeability is an important feature of biological cells that can be used as an indicator of a cell’s condition and death [2,3]. Nuclear Magnetic Resonance (NMR) diffusion imaging is a useful tool in studies on membrane permeability because it is sensitive to molecular motion. It has been shown by Mitra et al. that the time-dependent diffusion coefficient in the slow regime, i.e., when *t* ≪ *R*^2^*D*_0_^−1^, where *t* is a time of diffusion observation, *R* is a cell radius and *D*_0_ is a self-diffusion coefficient, is independent of microgeometry and membrane permeability [4] and can be applied to the determination of a self-diffusion coefficient of a liquid confined in a compartment with a specific Surface-area-to-Volume ratio, *S*/*V*.

Many works concern the application of a Pulsed Field Gradient (PFG) for studies on water dynamics in biological cells (e.g., [4,5,6,7,8,9,10,11] and references therein). However, in studies of porous systems with very small pores containing fluids whose transverse relaxation time is very short (<1 ms), the usage of the PFG technique may be problematic, mainly due to time limitations concerning the adjustment of sequence parameters. Fischer and Kimmich [12] discussed the problems associated with applying PFG and presented the method of a secondary stimulated echo for measuring the self-diffusion coefficients of polymers by using a constant gradient. The application of a constant gradient was also presented by Rata et al. [13] for the determination of self-diffusion coefficients in water-saturated sandstone cores.

A widely used model of diffusion in biological systems is the two compartmental model in which the exchange of water between the two pools occurs after a certain time, the so-called residence time or lifetime. An assumption about the two fractions making a contribution to the signal is sufficient for moderate gradient strengths and time parameters in pulsed techniques. This is due to the fact that intra- and extracellular space sizes are comparable to the diffusion length scales obtainable by systems with the mentioned features. This limitation may be overcome by the application of constant, very strong gradients. As we will show in this work, by using a constant gradient strength of 24 Tm^−1^ and Stimulated Spin Echo (SSE) sequence, it is possible to explore structures with length scales lower than 1 μm and self-diffusion coefficients significantly smaller than bulk water. In order to do so, a three-compartmental model was used to determine self-diffusion coefficients which were later analyzed in regard to their dependency on time. Admittedly, three compartments were applied earlier by Stanisz [14] to characterize diffusion in a bovine optic nerve, but one compartment was associated with extracellular space, while the other two with intracellular spaces with different geometries. On the other hand, Schoberth [8] studied small prokaryotic cells with sizes below 1 μm (*Corynebacterium glutamicum* bacteria having a diameter of 0.7 μm), but the PFG technique allowed him to measure intracellular water on the border between the localization and the motional averaging regime.

To the best of our knowledge, we applied the three-compartmental model of diffusion for the first time, where one of the compartments is significantly smaller than 1 μm and associated with cellular organelles. We will also show that, depending on the diffusion regime, the time-dependent diffusion coefficient will provide information on the biophysical properties of different structures. For this reason, when using an extremely strong diffusion gradient, three compartments cannot be rigidly assigned to specific spaces over the entire diffusion time range, as in the case of two-compartmental analysis.

## 2. Materials and Methods

### 2.1. Sample of a Model Cellular System

Fresh baker’s yeast (*Saccharomyces cerevisiae*; Lesaffre Polska S.A., Wołczyn, Poland) was purchased from a local market. Water content in the sample was equal to 24% of the total weight. The yeast was placed in a petri dish in its original form and its temperature was successively controlled. The experiments were conducted after ~1 h, when the yeast’s temperature was equal to the ambient temperature of 25 °C. The ambient temperature was maintained at a constant level. A referential measurement of a bulk water sample yielded a bulk water coefficient *D_bulk_* = (2.403 ± 0.044) × 10^−9^ m^2^s^−1^.

### 2.2. System

The ^1^H NMR diffusion measurements were performed on a Magritek Mobile Universal Surface Explorer (MoUSE; Magritek, Aachen, Germany). This device is constructed with the application of permanent magnets and allows measurements in a stray field. The construction scheme is presented in Figure S1. The magnetic field gradient is constant, directed perpendicularly to the surface of the table (marked as T in the Figure S1) and has an amplitude of 24 T/m. A slice can be chosen by an adequate movement of the high precision lift below the magnets. Due to the constant gradient, radio-frequency (RF) pulses can only excite a given slice of a sample at a chosen depth, compared to PFG techniques in which the whole sample is excited. All experiments were performed at 2.5 mm from the table, which was the depth in the lower half of the yeast. The slice thickness achieved for echo times (TEs) used in our study (from 0.04 ms to 1.2 ms) was equal to 200 μm.

### 2.3. Experiments

Diffusion was measured using the SSE pulse sequence (Figure S2). Time intervals *τ* and *t_m_* are analogues of *δ* and Δ in the PFG techniques and denote for gradient duration and gradients separation time, respectively. To register an echo attenuation for a particular diffusion time in PFG, the gradient’s amplitude is usually altered. Since our system operates at a constant gradient, attenuation was obtained via changing *τ* in 20 steps from *τ_min_* = 0.02 ms to *τ_max_* = 0.6 ms. The range of *τ*, as well as other parameters of the protocol, was kept the same for all the mixing times used in the experiments. Diffusion was measured for 15 *t_m_* = 0.2–40 ms, which enabled the achievement of *b*-values from the range of 0–5.97 × 10^11^ sm^−2^. The values of the parameters of the SSE pulse sequence are summarized in Table S1. The normalized echo attenuation for SSE is given by [15]:

(1) $$\frac{E}{E_{0}}\;=\;\exp\left(-\gamma^{2}G^{2}\tau^{2}\left(t_{m}\;+\;\frac{2}{3}\tau \right)D-\frac{2\tau }{T_{2}}-\frac{t_{m}}{T_{1}}\right),$$

where *γ* (T^−1^s^−1^) is gyromagnetic ratio, *G* (Tm^−1^) is gradient strength and *T*_1_ and *T*_2_ are the longitudinal and transverse relaxation times, respectively.

*T*_1_ and *T*_2_ relaxation times were obtained from *T*_1_ and *T*_2_ distributions. The *T*_2_ relaxation curve was acquired with the application of a Carr-Purcell-Meiboom-Gill (CPMG) pulse sequence for the following parameters: *TE*/*RD* = 40 μs/6200 ms, number of echoes = 4096, *NoS* = 256. *T*_1_ was measured with the application of a saturation recovery sequence using *TE*/*RD* = 40 μs/3500 ms, number of echoes = 2048, *NoS* = 16.

### 2.4. Models for Diffusion in Cellular System

**Figure 1:**
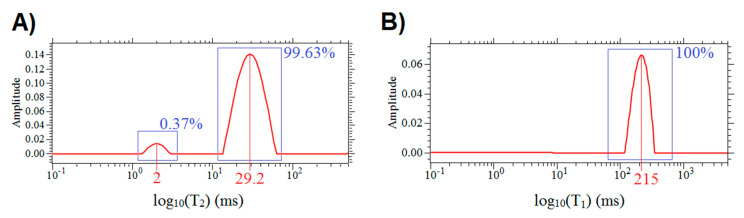
*T*_2_ (**A**) and *T*_1_ (**B**) distributions of a fresh yeast sample. Contributions of peaks to the whole distribution are presented in percentages.

Three models of diffusion in yeast were tested on the collected data: a two-compartmental (Model 0 when fitted in the full range of *b*-values, Model 0B when fitted in a given interval of *b*-values), a two-compartmental with an intercept (Model 1) and a three-compartmental model (Model 2). All of the models were fitted to the data in OriginPro2018b software. The overall formula for the echo attenuation in the sample for a particular *t_m_* is:

(2) $$\frac{E}{E_{0}}\;=\;\left(e^{-\frac{2\tau }{T_{2}}}\cdot e^{-\frac{t_{m}}{T_{1}}}\right)\cdot \sum_{i=1}^{n}p_{i}\cdot e^{-\left(\gamma^{2}G^{2}\tau^{2}\left(t_{m}\;+\;\frac{2}{3}\tau \right)\right)D_{i}}\;+y_{0},$$

where *T*_1_ and *T*_2_ relaxation times were taken from the distributions shown in Figure 1, *i* is *i*-th compartment in the sample, *n* is the number of compartments, *E* is the echo amplitude for the given *τ* and *t_m_* and *E*_0_ is the echo amplitude for the minimal *τ* and *t_m_* [13], *p_i_* is the molar fraction of *i*-th population and $\sum_{i=1}^{n}p_{i}=1$, *D_i_* (m^2^s^−1^) is the apparent diffusion coefficient in the *i*-th compartment, and:

(3) $${\left(\gamma G\tau \right)}^{2}\left(t_{m}+\frac{2}{3}\tau \right)\;=\;b,$$

which is a diffusion weighting factor in units (sm^−2^). *y*_0_ is the intercept and is equal to zero for Model 0, Model 0B and Model 2.

### 2.5. Time-Dependent Diffusion Coefficient (TDDC)

The multi-compartmental model of diffusion in yeast used further in this paper was based on images obtained from Transmission Electron Microscopy (TEM) or Scanning Electron Microscopy (SEM) reported in the literature (e.g., [16]), where at least one region of a significant size is visible, regarding nucleus, vacuoles, mitochondria or cell wall. Each of these regions is characterized by the self-diffusion coefficient dependent on diffusion time, which for *t_m_* ≫ *τ* is equal to *t_m_*, and will be hereafter called the time-dependent diffusion coefficient (TDDC). TDDC on the log-log scale evinces a characteristic S-shape with three distinct regions (Figure S3): I—free diffusion, II—restricted diffusion (localization regime) and III—hindered diffusion (motional averaging regime). Region I is described by Mitra’s relation [17]:

(4) $$D_{i}\left(t_{m}\right)=D_{0i}-\frac{4}{3W\sqrt{\pi }}\cdot \frac{S_{i}}{V_{i}}\cdot D_{0i}{}^{\frac{3}{2}}\cdot \sqrt{t_{m}},$$

where *W* is the number of space dimensions in which diffusion occurs, $D_{0i}\left(m^{2}s^{-1}\right)$ is the bulk self-diffusion coefficient in *i*-th compartment for *t_m_* → 0 and $\frac{S_{i}}{V_{i}}\left(\frac{1}{m}\right)$ is the Surface-area-to-Volume ratio. In this region, diffusion signal is independent of microgeometry, meaning that all structures with similar *D*_0_ contribute to one, *i*-th compartment. The arrow-marked region in II (Figure S3) can be described by the Einstein-Smoluchowski equation [11]:

(5) $$\langle Z_{i}^{2}\rangle \;=\;2dD_{i}t_{m},$$

where $\langle Z_{i}^{2}\rangle \;\left(m^{2}\right)$ is the root-mean-square displacement of water molecules during *t_m_* and *d* is the number of dimensions. This equation concerns diffusion in a non-permeable confinement. In reality, the power of diffusion time may be slightly higher than −1 [18] due to the permeable boundaries, i.e., cell membranes [10]. In this region, the diffusion coefficient is dependent on the microgeometry, which means that *i*-th compartment will be composed of structures having similar sizes. In the limit: $\tau \ll \frac{R_{i}^{2}}{D_{0i}},\;t_{m}\gg \frac{R_{i}^{2}}{D_{0i}}$, the root mean square displacement can be expressed as ([6] and references therein):

(6) $$\langle Z^{2}\rangle =\frac{2}{2+d}\cdot R^{2},$$

where *R* (m) is a half distance between boundaries (e.g., radius of sphere). *d* is, again, the number of dimensions, and in our case *d* = 1.

### 2.6. Simulations

In order to determine the contribution of each compartment to the signal attenuation during *t_m_*, Monte Carlo Random Walk (RW) simulations were conducted in the cell, in which random walkers reflected diffusing particles. Simulations were conducted in MATLAB (R2019b) (Natick, MA, USA, The MathWorks Inc.). The modeled two-dimensional (2D) geometry consisted of a cell wall, nucleus, mitochondrion, vacuole and intracellular space, in which we assumed completely reflecting boundaries. A non-exchangeable system was assumed based on the lifetimes reported in the literature for yeast (see Section 4.5). Particle jump duration was set to be *t_s_* = 5 μs (step length was dependent on the self-diffusion coefficient, *D*_0*i*_, $\sigma \;=\;\sqrt{4D_{0i}t_{s}}$). Number of RW steps depended on *t_m_* based on the relation *N* = *t_m_*/*t_s_* and was in the range of 40–8000, while the number of particles was equal to 5000. From the root-mean-square displacements of each particle the mean displacements *r_m_* in compartments during *t_m_* were calculated. By using *r_m_*(*t_m_*) and Equations (4) and (5), apparent diffusion coefficients were calculated and plotted versus *t_m_*. There was a certain time *t_m_* at which *D_i_* from Equations (4) and (5) were equal. To the left of this point we used *D_i_* from Equation (4), while to the right, we used *D_i_* from Equation (5). From these *D_i_*(*t_m_*) signal attenuations, *E*/*E*_0_(*b*) in each compartment was simulated using the Stejskal-Tanner equation, by the calculation of *b* for a priori taken *E*/*E*_0_(from 1 to 0.01).

### 2.7. Permeability

In the absence of osmotic gradients, no net water flux is observed during transmembrane water molecule exchange. In this case, water exchange is described by the diffusional membrane permeability, *P_d_*, which for spherical compartments can be calculated as:

(7) $$P_{di}=\frac{R_{i}}{3\tau_{i}},$$

where *τ_i_* is the lifetime of water in *i*-th compartment. It can be determined from the curve fitting to the time-dependent molar fractions, given by:

(8) $$p_{i}=p_{0i}\cdot e^{-\frac{t_{m}}{\tau_{i}}},$$

where *p*_0*i*_ is the compartmental molar fraction for *t_m_* → 0^+^ and represents the normalized (to the volume of the whole sample) volume of the compartment.

## 3. Results

The collected data in the diffusion experiments are a set of echo attenuations for different mixing times. It contains information about TDDC and molar fractions for all the modeled regions, which are treated as fitting parameters.

### 3.1. Relaxation Times

The *T*_1_ and *T*_2_ time distributions of a sample obtained in NMR-MoUSE are shown in Figure 1. *T*_2_ s are apparent *T*_2_ relaxation times, decreased compared to the bulk fluid’s *T*_2_, due to the high diffusion impact resulting from the application of the high gradient. However, these *T*_2_ s are visible in the diffusion experiment in the same system. *T*_2_ times of the peaks were found to be equal to 2 ms and 29.2 ms, while *T*_1_ = 215 ms. The peak with *T*_2_ = 2 ms can be neglected due to a very low contribution. Peaks with *T*_2_ = 29.2 ms constitute 99.63% of a whole relaxation signal, meaning all cellular structures have similar relaxational properties, and come from the free fluids in the cell system. We can assume that in the diffusion experiment, the attenuation of a signal due to relaxation will be associated with *T*_2_ = 29.2 ms and *T*_1_ = 215 ms.

### 3.2. Choosing the Appropriate Diffusion Model

**Figure 2:**
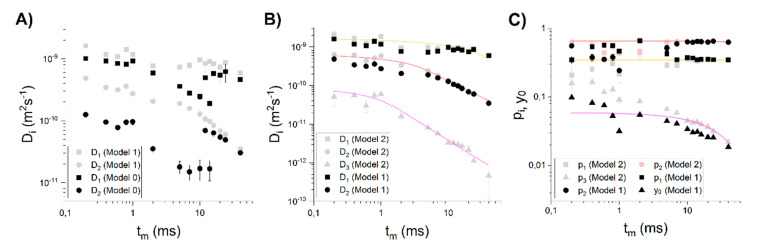
(**A**) Comparison of results from the fitting of the model 0 and 1. (**B**) Comparison of results from the fitting of the Model 1 and 2. The lines are fitted to *D_i_*(*t_m_*) from model 2 with the application of (4) and (5). (**C**) Molar fractions obtained from model 1 and 2 with fitted lines for *t_m_* = 10−40 ms.

First, we adapted the most common, two-compartmental model (model 0, Figure 2A), in which two non-exchangeable (*t_m_* much smaller than water lifetimes reported for yeast) regions are associated with extra- and intracellular water. However, even for the low *t_m_*s, model 0 does not fit the data well and for higher *t_m_*s, it does not even converge on the data points for *b* values higher than approximately 1 × 10^10^ sm^−2^. To see whether this situation is due to systematic errors (resulting for example from inequality of *T*_2_s in the compartments [5]), a two-exponential model with intercept (model 1) was adapted (Figure 2). Model 1 indeed fits the data better for the low *t_m_*s, but the intercept *y*_0_ consisted of as much as 10% of a signal. Additionally, molar fractions *p_i_* from model 1 did not exhibit an exponential decay; rather, they are randomly distributed. Furthermore, *y*_0_ revealed a very strong discontinuity at *t_m_* between 1 and 2 ms (Figure 2C). A rapid increase of *p*_2_ in connection with a rapid drop of *p*_1_ values at *t_m_* = 2–5 ms is in accordance with the *D*_1_ and *D*_2_ discontinuity point (Figure 2B). It looks like *D*_2_ was composed of two decaying regions II (marked with arrow in the Figure S3) separated at *t_m_* = 2−5 ms. In case of *D*_1_, the points start to decay even for the very small *t_m_*s and then remain constant. Considering that this component is associated with extracellular space, it is unlikely that region II is observed for very low *t_m_*s, meaning that diffusion is restricted. Due to its size, extracellular space enables water to diffuse freely for a relatively long time. On this basis, we can suspect that the third compartment of slowly diffusing water is visible in the experiments. For very low *t_m_*s (<1 ms), it gives almost no attenuation to the total signal and appears as a high intercept *y*_0_, while for high *t_m_*s, it will have high impact on the attenuation curve (especially for the second half of *b*-values for which the first two compartments are rather fully attenuated, but the signal is still detectable).

The first qualitative analysis shows that three-compartmental is the most preferable. Assuming that all of the three compartments will contribute to the signal attenuation for all *t_m_*s for a given *b*-values range, model 2 was fitted. Next, a quantitative comparison of the model 1 and 2, was made. The models were compared via statistical tests—Akaike’s (AIC) and Bayesian Information Criterion. Figure S4 shows that for almost all cases, there is a higher probability (Akaike’s weight) that model 2 is a true model. For extreme mixing times, Akaike’s weights are higher for model 1, but BIC is inconclusive. These findings suggest that signal attenuation depends on the three components simultaneously, only for some *t_m_*s, while for the very short and long *t_m_*s signal is attenuated mostly due to diffusion in two compartments. Considering that *D_i_*s for *t_m_* → 0 are of the order of ~1 × 10^−9^, ~1 × 10^−10,^ ~1 × 10^−11^, it is supposed that for short *t_m_*s, the signal is attenuated mostly by the fastest and the intermediate components, while for long *t_m_*s by the intermediate and slowest ones.

To find out for which diffusion weighting, *b*, the signal from the fastest component is completely attenuated and for which *b* the slowest one starts to contribute to the total signal attenuation, we simulated diffusion signal behavior *E*_1_/*E*_01_, *E*_2_/*E*_02_ and *E*_3_/*E*_03_ in compartment 1 (the fastest diffusion, extracellular water), 2 (intermediate diffusion, cytoplasm) and 3 (the slowest diffusion, different cellular organelles), respectively. Then, *E*_1,2,3_/*E*_0_ was analyzed. However, a particle’s diffusive behavior will be different in different geometries (i.e., planar, spherical or cylindrical). For this reason, compartments had to be matched with a concrete water pools and their geometry characterized by size, shape, molar fractions and self-diffusion coefficients. The preliminary information about compartments was taken from the TDDCs resulting from the fitting of model 2.

### 3.3. Relating Compartments with Cellular Structures

Firstly, the three compartments were characterized by the approximate self-diffusion coefficients *D*_0*i*_ and sizes *R_i_*. Considering that *D*_1_ is rather constant and similar to *D_bulk_*, the first compartment was assigned to the extracellular water assumed earlier, with *D*_01_ ≈ *D_h_*_1_ ≈ 1 × 10^−9^ m^2^s^−1^. Since *D*_1_ ≈ const., *E*_1_/*E*_01_ (*b*) obtainment did not require the simulation of random walks in extracellular space in order to determine *D*_1_(*t_m_*) from root-mean-square displacement. Intermediate and slow components show typical S-shape decay (Figure S3) and regions I and II can be recognized (Figure 2B)). Equations (4) and (5) were fitted to the regions I and II, respectively. For the diffusion coefficient of cytoplasm, *D*_2_, the two curves merge perfectly at *t_m_* = 5 ms. *D*_02_ obtained from the fitting of (4) was equal to 0.676 ± 0.041 × 10^−9^ m^2^s^−1^, while compartment size *R*_2_ from the fitting of (5) was equal to 2.79 ± 0.11 μm. The reason why the second’s compartment size is better determined from (5) than from the *S*_2_/*V*_2_ will be explained in Section 4.4. For the slowest component, (4) was fitted in the range 0.2 ms to 2 ms, i.e., the first point after the plateau in region I, which delivered *D*_03_ = 0.095 ± 0.011 × 10^−9^ m^2^s^−1^. A linear part of TDDC is clearly visible in the range of *t_m_* = 0.8–40 ms. The lines fitted in the region I and II merge satisfactorily at *t_m_* ≅ 1.5 ms. As in the case of the intermediate component, an allometric function was fitted that yielded a compartment size *R*_3_ = 0.277 ± 0.048 μm.

Molar populations, *p_i_*, in the range of 0.2–7 ms do not lie on the fitted lines. For *t_m_* < 10 ms they are chaotically distributed, which results from the application of model 2 in the whole range of *b*-values and *t_m_*. *p_i_*s tend to decrease mono-exponentially from 10 ms for all compartments, where (8) was fitted. The poor fit of a mono-exponential function to the *p*_3_ suggests that *p*_3_(*t_m_*) dependency has a bi-exponential character. However, due to the erroneous *p*_1_ and *p*_2_, the short-time behavior of the *p_i_*s form model 2 is not analyzed, and therefore, there is no need for bi-exponential fitting. Mono-exponential fitting delivered approximate equilibrium molar fractions *p*_01_ = 0.3513 ± 0.0073, *p*_02_ = 0.6501 ± 0.0089 and *p*_03_ = 0.0648 ± 0.0037 for compartments 1, 2 and 3, respectively. The sum of *p*_0*i*_ > 1, which is due to the fitting three components for high *t_m_*. This, together with short/intermediate-time behavior, is further explained in the next subsection.

**Figure 3:**
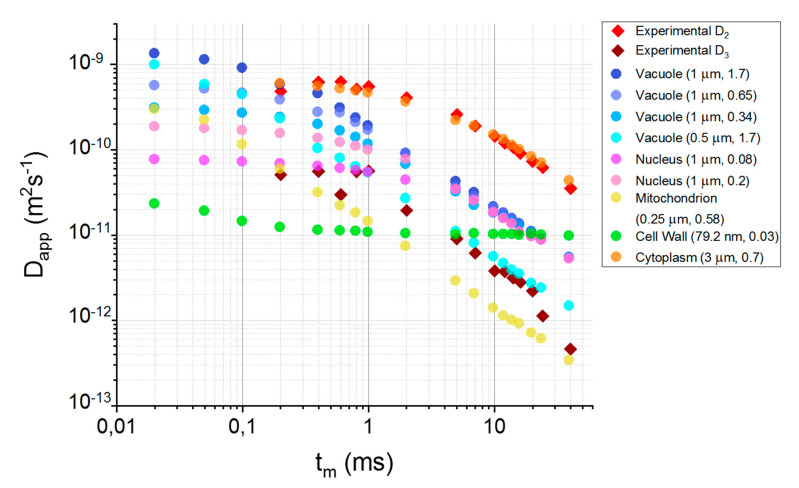
Time-Dependent Diffusion Coefficients (TDDCs) obtained in the experiments with comparison to simulations conducted for cytoplasm and cellular organelles; the legend presents the name of the structure with its size and literature value of self-diffusion coefficient in units ·10^−9^ m^2^s^−1^ put in the bracket.

Identification of cellular structures associated with the third compartment was based on the comparison of *R*_3_, *p*_03_ and *D*_03_ with literature values shown in Table 1. Taking into consideration that *p*_03_/*p*_02_ ≈ 0.1, ratios of total volume of structure and intracellular volume, *f*, as well as *f*·*p*_02_ are presented. Most of the structures are characterized by the self-diffusion coefficient similar to that of the second component—cytoplasmic water. *D*_03_ is of the same order of magnitude as *D*_0_ of a cell wall and nucleus. Considering the determined *D*_03_ and *R*_3_, it is possible that the third compartment contains an averaged signal coming from several structures. RW simulations were conducted separately for cytoplasm, nucleus, mitochondrion, vacuole and the cell wall. Calculated TDDCs are presented in Figure 3.

### 3.4. Simulation of the Diffusion Behavior in Cells

**Figure 4:**
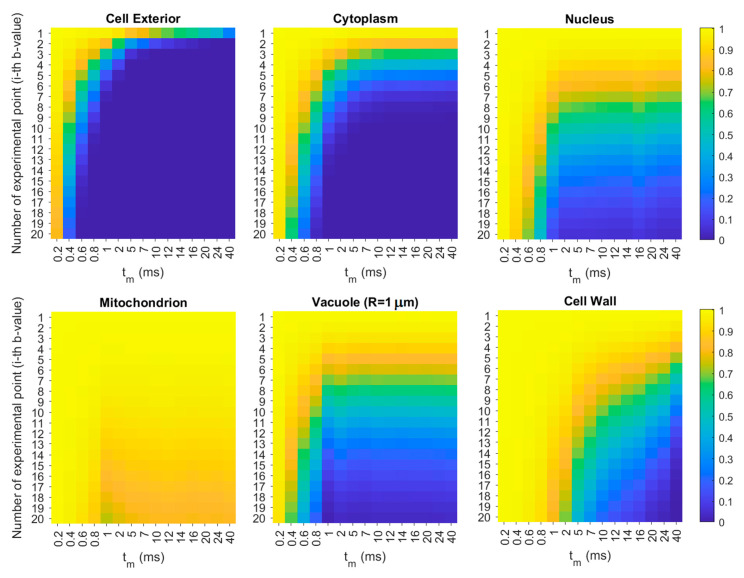
Simulated signal intensities in the given cell structure and extracellular space (*E_j_*/*E*_0*j*_, where *j* corresponds to the given pool (Cell Exterior, Cytoplasm, Nucleus, Mitochondrion, Vacuole and Cell Wall, while *E_j_* and *E*_0*j*_ are signals registered with and without diffusion weighting) dependent on *b*-value and mixing times, *t_m_*.

**Figure 5:**
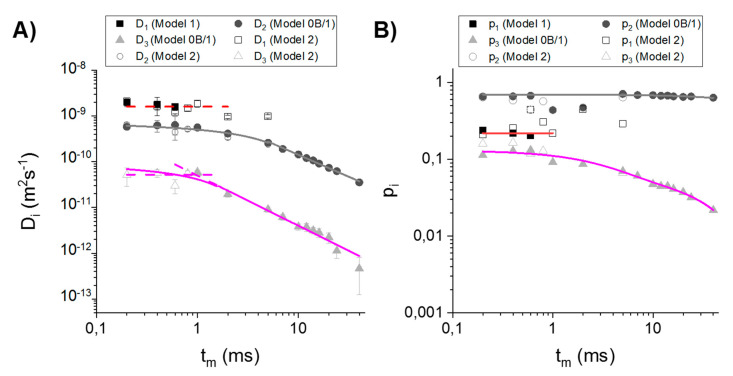
TDDCs (**A**) and molar fractions (**B**) of the three compartments obtained from the fitting of model 0B in the range *t_m_* ≫ *τ* for which *t_m_* is equal to diffusion time. Additionally, results from the fitting of model 2 are presented.

Diffusion coefficients simulated for different water pools in the sample are shown in Figure 3. In the next step, with the use of these coefficients, we simulated signal intensities, which are shown in Figure 4. As we can see, cellular structures do not significantly attenuate the total signal for *b* < *b*_16_ (null attenuation from the third compartment), for *b* < *b*_13_ and for *b* < *b*_8_ for *t_m_* = 0.2, 0.4 and 0.6 ms, respectively. In these ranges, two-compartmental model 1 can be applied instead of the more parametric model 2, where the intercept *y*_0_ reflects non-attenuating *p*_3_. However, for *t_m_* = 0.6 ms, seven points seem to be too few (erroneous fitted parameters), thus, one of the parameters had to be constrained (in our case we arbitrarily chose *D*_1_). The signal from extracellular space is rapidly attenuated for *t_m_* > 2 ms. This signal is virtually null for the fourth, third and the second *b*-value for *t_m_*s equal to 5–7 ms, 10–24 ms and 40 ms, respectively. This means that the first component obtained from the fitting of model 2 to the experimental attenuation curves (Figure 5A) was fitted to only several points. It is highly probable that for higher *t_m_*, the first exponent was partly fitted to the points for which in practice the attenuation came from the second compartment. As a result, fitting could deliver *D*_1_, but also *p*_1_, which are associated partly with the first compartment and partly with the second (i.e., averaged *D*_1_ and *p*_1_). In consequence, *D*_1_ is underestimated, while *p*_1_ overestimated (therefore, the sum of *p*_0*i*_ in the previous subsection was higher than 1). Hence, for *t_m_* > 2 ms, it was reasonable to exclude the first 2, 3 or 4 points from the data set and to perform the less parametric, two-exponential fitting to the signal coming only from the second and the third compartment. This approach is called model 0B fitting, which delivered the experimental *D*_2_ and *D*_3_ values shown in Figure 3.

Attenuation of the signal coming from the cytoplasm is visible in the full range of *t_m_*. For *t_m_* > 1 ms, the signal from cytoplasm is null in the second half of experimental data points. For these points (*b*-values), the diffusion signals are still intensive for the nucleus, mitochondrion, vacuole and cell wall that comprise the third compartment. Hence, the two-exponential fitting seems to be a good approach, since the number of fitted parameters in each half is a few times lower than the number of data points.

In Figure 3 we can see that the experimental *D*_2_s coincide almost perfectly with the *D*_2_s obtained in the simulations, which confirms that the second compartment is associated with cytoplasm. Figure 3 also shows apparent diffusion coefficients obtained in the simulations for the cell’s structures with comparison to the experimental third component, *D*_3_. Considering the amounts of signal in the organelles (Figure 4) and the alignment of the experimental *D*_3_ with respect to the simulated TDDCs of organelles (Figure 3), the third compartment is probably associated with all of these organelles; however, in the experiment we can observe the averaged signal, *D*_3_. Moreover, the contribution of each organelle will depend on their signal’s attenuation rate for a given *t_m_*.

### 3.5. Extraction of Compartmental Characteristics from TDDCs

Based on Figure 4, it was possible to approximately identify the *b*-values for each *t_m_*, for which the signal from the first compartment is attenuated. Fitting the sum of two exponents in this range was called model 0B fitting. The choice of this approach was explained in the previous section. The sample’s microgeometry was characterized based on TDDCs and molar fractions decays obtained from the fitting of model 0B for *t_m_* > 2 ms (Figure 5). This approach delivered compartment sizes *R*_2_ = 2.252 ± 0.053 μm and *R*_3_ = 0.277 ± 0.058 μm. The equilibrium of molar fractions calculated from interpolation of lines obtained from the fitting of (8) were *p*_01_ = *const.* = 0.2188 ± 0.0075, *p*_02_ = 0.6959 ± 0.0052, *p*_03*a*_ = 0.070 ± 0.021 and *p*_03*b*_ = 0.060 ± 0.028 (yielding total *p*_03_ = 0.130 ± 0.035), while residence times *τ*_2_ = 390 ± 56 ms, *τ*_3*a*_ = 3.3 ± 2.0 ms and *τ*_3*b*_ = 39 ± 28 ms. In the case of extracellular water, it was not possible to genuinely determine water lifetimes from the (8) for *t_m_*s for which the extracellular signal is not completely attenuated (*D*_1_(*t_m_*) and *p*_1_(*t_m_*) are approximately constant there—see the red lines in Figure 5). In practice, using model 0B over model 2 allows a more accurate determination of *D*_2_ and *D*_3_, which results in slightly different compartments sizes, as well as *p*_2_ and *p*_3_, which leads to the obtainment of residence times that are up to two times higher.

## 4. Discussion

NMR diffusometry was performed for several mixing times, which were much smaller than the lifetimes of water molecules in the extra- and intracellular space reported in the literature for yeast [5,11,38]. Based on this knowledge, we assumed that there is no exchange between the interior and exterior of the cell during the experiments. The very strong magnetic field gradient imposed very large *b*-values (Table S1) that significantly exceeded 5 × 10^9^ sm^−2^, above which it is said that the intracellular signal can be predominantly detected [39]. Such a strong diffusion weighting led us to suppose that there was another, very slowly diffusing component to be detected. We qualitatively compared the three diffusion models in yeast: two-compartmental (or Kärger model [40], which is a simple model of diffusion in two compartments between which water exchanges; model 0), two-compartmental with intercept (model 1) and three-compartmental (model 2). Model 0 was not satisfactory, especially for *t_m_*s for which *b* values exceeded ~$1\times 10^{10}$ sm^−2^, and was excluded from further analysis. In the next step, model 1 and model 2 were qualitatively as well as quantitatively compared via AIC and BIC (Figure S4). Inconclusive results for extreme *t_m_*s indicated that in our experiments different components influence signal attenuation depending on the *b*-value range. Identification of those ranges was possible after RW simulations (Figure 3 and Figure 4) with the application of the literature values of sizes and self-diffusion coefficients of yeast’s cellular structures (Table 1). Knowledge of the approximate *b*-values for which extracellular water is attenuated allowed us to apply the simpler, two-compartmental model (model 0B) associated with the cytoplasm and cellular organelles.

### 4.1. Violation of the t_m_ ≫ τ Condition

TDDC analysis requires the fulfillment of the Short Gradient Pulse (SGP) or in other words- *t_m_* ≫ *τ* condition. In our experiments, the condition was violated for some experimental points *τ_i_* for *t_m_* < 1 ms, where *t_m_* was not equal to the real diffusion time, $t_{d}=\left(t_{m}+\frac{2}{3}\tau \right)$ and *t_d_* was not constant during the acquisition of the single signal attenuation curve. However, the fitting lines in the region I and II connect almost perfectly, while the points simulated based on the fitted parameters coincide with the experimental data. Mean error in the short-time regime was equal to ~11% (*t_m_* = 0.2–1 ms). It is possible that taking into account all of the cellular organelles in the cellular geometry modeling during the simulations of RW would reduce the mean error. More intracellular restrictions would cause shorter root-mean-square displacements and, in consequence, slightly higher *D*_2_s, like those obtained experimentally. More ambiguous values can be observed for the *p_i_*(*t_m_*) dependence, which is a smooth, single exponential dependence only above the short-time limit. It is hard to say whether the evident two-exponential *p_i_*(*t_m_*) dependency or the points not lying on the fitted lines result from the erroneous fit, physiological processes (namely diffusive exchange) in the system or the non-compliance of the time-related requirements ($t_{d}\neq const.$). Based on these observations we can say that under conditions of *t_m_* ≫ *τ* violation causing $t_{d}\neq const.$, distortions are visible for the molar fractions, while for *D_i_*(*t_m_*) they are minor.

### 4.2. Comparison of the Diffusion Models

As shown in the Results section, three compartments cannot be characterized in the full range of *b*-values by using model 1, because the observations show that the 2% drop of signal coming from an organelle with a molar fraction of several percent is already visible in the experiment with high SNR (in this work—128 scans). The intercept values in model 1 were equal to 10% of the total signal, which is a significant amount considering the capabilities of a NMR-MoUSE system to detect very slow (*D*~10^−15^ m^2^s^−1^), low-populated components (even 1% of the total population according to Williamson [41]). Model 2, which delivers smooth TDDC, but biased parameters, can be applied only for the determination of approximate values of parameters. The results suggested that signal attenuation results from the three different components depending on the *t_m_* range—fast and intermediate for short *t_m_*s (low *b*-values) and intermediate and slow for longer *t_m_*s (high *b*-values), forcing interval fitting. Fitting the simpler model 1 in the interval of low *b*-values and low *t_m_* yielded more accurate values of *D*_1,2_ and *p*_1,2_ associated with extracellular water and cytoplasm. The second interval included attenuation due to the diffusion in cytoplasm and cellular structures and embraced *b*-values for which extracellular signal is fully attenuated. In this interval, the fitting of the simpler model 0B was beneficial and delivered more accurate parameters in comparison to model 2 as mentioned in Section 3.5.

Interval fitting using model 1 and model 0B over model 2 was advantageous due to the less fitted parameters and reduced risk of fitting a component to the points, for which this component is attenuated in reality. The percentage differences between the fitted parameters from the models 2 and 0B were equal to 0.1–21.3% with a mean of 7.7%, 0.7–22.6% with a mean 9.0%, 0.1–37.9% with a mean of 10.1% and 0.1–10.2% with a mean of 7.6% for *D*_2_, *D*_3_, *p*_2_ and *p*_3_, respectively. In the case of parameters obtained from model 2 and 1, the percentage difference was equal to 4.9–31.3% with a mean of 14.7%, 0.1–42.4% with a mean of 17.5%, 14.7–54.6% with a mean of 28.1%, 2.4–52.0% with a mean of 28.1% and 0.1–10.2% with a mean of 7.6% for *D*_1_, *D*_2_, *p*_1_, *p*_2_ and *p*_3_, respectively. The analysis shows that model 2 can be incorporated into the signal attenuation curve fitting in the whole range of applied *t_m_*s and *b*-values yielding reasonable outcomes, especially when it is unable to identify the attenuation rate or signal amount associated with a particular component in a given range. A significant improvement due to the application of simpler models was observed particularly in the short-time regime and especially for molar fractions. From an analytical point of view, the improvement relies on the fact that by the elimination of 2/3/4 experimental points, the number of points per number of fitted parameters increases from 3.33 up to 4.75, while the total number of points, *N* (= 18, 17, 16), is much higher than the number of fitted exponents.

### 4.3. Characterization of the Compartments Based on TDDCs

Based on the TDDCs, compartment characteristics were obtained in Section 3.4. Additional information can be inferred from Figure 3 and Figure 4. The first compartment visible in Figure 5 is associated with the extracellular water. For *t_m_* = 0.2–1 ms, the mean diffusion coefficient *D*_01_ = 1.64 ± 0.15⋅10^−9^ m^2^s^−1^ (Figure 5A)), which is similar to the self-diffusion coefficient 1.6 × 10^−9^ m^2^s^−1^ reported for extracellular fluid [5]. Extracellular space size cannot be accurately determined from (4) (*V*_1_/*S*_1_ ≅ *R*_1_ = 2.84 ± 0.36 μm), because there are very few data points for which the extracellular signal is still detectable, which means that the first exponent contains averaged information about the first and the second compartment and the obtained *D*_1_s are underestimated. It cannot also be successfully compared with reported sizes, due to the significantly lower water content (~24% compared to 80% water content in the work of Suh [11], who obtained extracellular space sizes equal to 15–20 μm). Nonetheless, *D*_1_ can be slightly time-dependent and *R*_1_ can be reduced, because fresh yeast’s extracellular space size is smaller than in commonly studied suspensions or sediments. Model 1 delivered *p*_01_ = 0.2188 ± 0075, which is similar to the theoretical and experimental molar fractions of extracellular space reported by Conway and Downey [42] equal to 0.26 and 0.22–0.24, respectively. On the other hand, they reported that extracellular space can be increased to 0.33–0.34 and seen as a thick cell wall. This is in accordance with our analysis.

The second and third compartments are associated with different structures depending on the *t_m_*. It is well-known that in the short-time regime (region I in Figure S3), the diffusion signal is independent of the microgeometry (free diffusion regime). Therefore, *D*_2_ and *D*_3_ in this regime will encompass all structures with a similar self-diffusion coefficient. In the short-time limit, the second compartment, *D*_2_, reflects the diffusion signal in the cytoplasm (*D*_0,*cytoplasm*_ equal to 0.5 × 10^−9^ m^2^s^−1^ [19], up to 1.0 × 10^−9^ m^2^s^−1^ [37]), vacuoles (if we assume a vacuolar size of 1 μm and *D*_0,*vacuole*_ equal to 0.34 × 10^−9^ m^2^s^−1^ [36] or 0.61⋅10^−9^ m^2^s^−1^ calculated based on the Stokes relation [19] using a vacuolar viscosity equal to 2.52 cP [43] or 1.7⋅10^−9^ m^2^s^−1^ [37]) and mitochondria (if we assume *D*_0*,mitochondrion*_ = 0.58 × 10^−9^ m^2^s^−1^ [23]; however, it is the apparent diffusion coefficient calculated for water-mitochondria suspension). *D*_3_ in the short-time limit can be connected with the nucleus (*D*_0*,nucleus*_ = 0.01–0.1 × 10^−9^ m^2^s^−1^), cell wall combined with cellular membrane (*D*_0,*CW&membrane*_ = 0.03 × 10^−9^ m^2^s^−1^) and mitochondrion (if we assume *D*_0*,mitochondrion*_ = 0.01–0.1 *D_bulk_*). This is well depicted in Figure 4, where experimental *D*_3_ in the short-time regime lies near the nuclear (light and dark pink dots), cell wall’s (green dots) and mitochondrion’s (red dots) TDDCs. The very low fraction of mitochondrion volume in relation to the whole cell’s volume suggests that its signal is of minor importance.

In the region II of TDDC (Figure S3), the diffusion signal becomes dependent on the confining geometry (localization regime). Thus, TDDCs will be associated with structures having similar sizes. First of all, *D*_3_ will no longer depend on the nucleus signal. This is due to the fact that the nuclear pore complexes are relatively large (~100 nm [44]) and molecules up to 20–40 kDa can diffuse freely through them [45]. The high permeability of the nuclear envelope leads to water residence time in the nucleus being very short (using the dependency of osmotic permeability versus permeant’s radius obtained on the basis of the values presented by Paine for oocytes [46], nuclear water residence time in the conditions of osmotic gradient is approximately 0.03 ms, while it is ~0.1–1 ms when considering that the diffusive permeability is 4–70 times lower than osmotic one [47]). Hence, the *t_m_* > 1 ms signal coming from the nucleus will already be mixed with the cytoplasm signal (motional averaging) and will contribute to the second compartment, *D*_2_. *D*_3_ above the short-time limit will mostly depend on the diffusion in vacuoles, but it is possible that their sizes are slightly smaller than those found in the literature (Table 1), which can be seen in Figure 3 (blue dots representing vacuolar radius equal to 0.5 μm). The transmission electron micrographs presented by Baba and Osumi [48] (for example Figure 5 therein) clearly show that vacuoles are significantly smaller than the nucleus and have a diameter of ~1 μm.

Incorporating theoretical *p*_01_ = 0.2 and sizes of structures determined in this study (the exception was the vacuole, for which we assume a radius of 0.5 μm), we estimated the expected molar fractions for each compartment. Based on the contributions to each compartment in the short-time limit, the determined fractions were equal to 0.69 and 0.11 for the second and third compartment, respectively. These values are in very good agreement with the experimental molar fractions obtained in the study. In the case of *p*_3_(*t_m_*), similarly to the first compartment, bi-exponential dependence can also be seen. Fitting the second component to the *p*_3_(*t_m_*) in the short-time regime delivers *p*_03_ = 0.129 ± 0.016, which is even closer to the theoretical value of 0.11.

### 4.4. Characterization of the Sample’s Microgeometry

The cell radius obtained from the fitting of (5) in region II in the study was *R*_2_ = 2.252 ± 0.053 μm, which is in very good agreement with the cell radius calculated from *S*_2_/*V*_2_ equal to 2.35 ± 0.40 μm. Small discrepancies may result from the effect of different surface-area and volume of the second compartment in the free and localization diffusion regime. Åslund reported a yeast cell radius of 2.48 μm [6], Tanner and Stejskal obtained a yeast cell diameter equal to 4.1 μm [9], while Cory obtained a radius of 5 μm [49] and Suh identified yeast cell radii equal to 2.3 ± 0.2, 3.0 ± 0.2 and 2.7 ± 0.2 μm for incubation times equal to 9, 24 and 48 h, respectively [11]. As we can see, our values are in the range of the abovementioned cell sizes. The difference between *R*_2_ obtained from the fitting of (5) and from the *S*_2_/*V*_2_ results from the fact that signal from nucleus in the short-time limit is associated with the third compartment, not the second one. A cytoplasm is characterized by the self-diffusion coefficient *D*_02_ = 0.692 ± 0.060 × 10^−9^ m^2^s^−1^, which is similar to the value for the intracellular self-diffusion coefficient reported in the literature for the two-compartmental model. For example, Tanner and Stejskal obtained a value of 0.68 × 10^−9^ m^2^s^−1^ [9], while Aslund obtained a value of 0.65 × 10^−9^ m^2^s^−1^ [6]. The slightly higher value may result from the SGP violation or from the fact that it is associated with relatively high diffusivity cytoplasm, not the whole cell interior.

The size of the third compartment in the short-time regime obtained in the study is a weighted mean size of the nucleus and cell wall as mentioned in Section 4.3. Assuming a literature value of the extracellular molar fraction *p*_01_ = 0.2 (and correspondingly *p*_02_ = 1–*p*_01_ = 0.8), the molar weights of cellular structures were determined. Applying these values in a short-time limit, we estimated the weighted mean size *R*_3_ = 0.422 μm, which is in a very good agreement with *R*_3_ = 0.415 ± 0.016 μm determined from *S*_3_/*V*_3_ from Mitra’s relation fitted to *D*_3_(*t_m_*). In the analysis of the region II of *D*_3_, the estimated *R*_3_ = 0.278 ± 0.040 μm, which reflects the weighted mean size of the cell wall and the average vacuolar size. Based on the work of Baba [48] and our results, we can assume that *S. cerevisiae* cells in the studied sample also contained vacuoles with a radius of 0.5 μm or less.

### 4.5. Diffusive Permeabilities

The diffusive permeability of the vacuolar membrane *P_d_*_3_ was determined based on (7) and was equal to 2.38 ± 0.66 μm/s. This value is within the range of the two limiting values of permeability for sphingomyelin/cholesterol and phosphatidylcholine/cholesterolbilayer membranes at 25 °C equal to 0.81 and 5.73 μm/s, respectively [38]. The diffusive permeability of yeast cell membrane *P_d_*_2_ determined based on (7) was equal to 1.93 ± 0.10 μm/s, which is very similar to 0.92 μm/s [38] and 0.185–1.35 μm/s [50]. Interestingly these values are relatively low compared to the value of 6.3 ± 0.6 μm/s estimated by Suh [11]. The same situation occurs for intracellular lifetimes reported to date, which differ among papers. Exemplary values are 0.833 s [38], 0.240 s, 0.450 s and 0.400 s [11], which are very similar to the value of 0.390 s determined in this work. All characteristic parameters for the model cell system used in the work are summarized in Table 2.

## 5. Conclusions

An NMR-MoUSE with a very high steady gradient (24 T/m) was used in the characterization of living cells by means of diffusion NMR. The three-compartmental model was tested and favored for the diffusion in the middle range of applied *t_m_*s. Accurate characterization of compartments was supplemented by random walk simulations and theoretical calculations supported by an extensive literature review. Complex analysis, including the theoretical behavior of diffusion and the analysis of biophysical processes in cells, was necessary to understand the physical results reflected by signal attenuations obtained in our system. This work shows that NMR diffusometry can be used to explore biophysical processes occurring far below the extra- and intracellular level. A very good level of agreement between the experimental and theoretical results proves that cellular organelles can be studied in terms of their biophysical properties by the application of NMR-MoUSE, something never previously achieved by diffusion NMR without the isolation of a given structure. Additionally, we presented the signal behavior depending on the SSE sequence parameters in the work, which can be used as a guide for choosing the appropriate values of *b*-values or *t_m_* for measurements oriented towards specific compartment studies.

In the MoUSE system, in which RF pulses are applied with a constantly present high gradient, the excited slice thickness is of the order of 100 micrometers. Considering this fact, detecting a signal from low-populated components such as water in nuclei is beneficial with regard to the small amount of samples that scientists often have at their disposal. To sum up, self-diffusion coefficients, sizes and molar fractions of extracellular water, cytoplasm and cellular structures can be obtained from the analysis of a time-dependent diffusion coefficient using single-sided NMR-MoUSE.

## Figures and Tables

**Table 1:** Literature values of parameters characterizing cellular structures in yeast cell; *N_s_*—number of structures in a single cell, *V_t_*—total volume calculated from geometrical calculations, *f*—total volume and intracellular space volume ratio, *D*_0_—self-diffusion coefficient of water in the structure found in the literature, *p*_02_—equilibrium molar fraction of intracellular water.

Cell Structure	Size (μm)	*N_s_*	*V_t_* (μm^3^)	*f*	*D*_0_ (×10^−9^ m^2^/s)	*f p*_02_
Whole cell	~3	1	113	--	--	--
Intracellular space (whole cell without CW)	2.82–2.92	1	93.9–104	1	~0.5–0.7 [5,6,9,19]	0.65
Nucleus	1 [20,21]	1	4.19	0.04	~0.01–0.1 [22], 0.23 (erythrocyte) [23], 0.04 (oligonucleotides) [24]	0.026–0.029
Cell Wall (CW) and cell membrane (combined)	0.0792–0.180 [25]	1	8.72–19.2	0.08–0.204	~0.03 (weighted mean of CW and cell membrane)	0.054–0.133
Cell membrane	0.0092 [26]	1	0.922–0.989	0.00989–0.00922	0.44 (water between lipid bilayer), <0.0006 (lipids) [27]	0.0062–0.0063
Cell Wall (CW)	0.070–0.1708 [28]	1	7.73–18.2	0.07–0.194	0.032 ± 0.014 (Carboxyfloresceine in Thale cress) [29]	0.048–0.126
Mitochondrion	0.25 [30,31]	2.3 [32]	0.151	0.0014–0.0016	0.58 (liver mitochondrion) [23], ~0.01–0.1 D_bulk_ [33]	0.00104
Vacuole	1 [34]	2.7 [35]	11.3	0.11–0.12	0.34 (Besidiomycete fungi at 20 °C) [36], 1.7 (apples) [37]	0.0704–0.0783

**Table 2:** Characteristic parameters for three compartments obtained in the study; *i* means the *i*-th compartment, *D*_0_ is a self-diffusion coefficient, *S*/*V* is the Surface-area-to-Volume ratio, *p*_0*i*_ is the equilibrium molar fraction of compartment, *τ* is the lifetime of water, *R_i_* is the compartment size, *P_d_* is the diffusive permeability of the compartment’s boundary.

*i*	*D*_0*i*_(×10^−9^ m^2^s^−1^)	*S_i_*/*V_i_* (μm^−1^)	*P*_0*i*_	*τ_i_* (s)	*R_i_* (μm)	*P_d_* (μm/s)
1	1.64 ± 0.15	–	0.2188 ± 0.0075	0.201 ± 0.039	–	–
2	0.692 ± 0.060	1.28 ± 0.22	0.6985 ± 0.0068	0.390 ± 0.056	2.252 ± 0.053	1.93 ± 0.10
3	0.095 ± 0.011	7.22 ± 0.28	0.070 ± 0.021	3.3 ± 2.0	0.277 ± 0.048	2.38 ± 0.66
			0.060 ± 0.028	0.039 ± 0.028		

## Supplementary Materials

The following are available online at https://www.mdpi.com/2073-4409/9/9/2124/s1, Section S1. Materials and methods, Figure S1: The construction scheme of the NMR-MoUSE device, Figure S2: The Stimulated Spin Echo (SSE) pulse sequence used in the experiment. Table S1: Parameters of the protocol used in diffusion measurements. Figure S3: Log-log plot of diffusion coefficient dependency on diffusion time, Section S2. Theory of model comparison based on Akaike’s (AIC) and Bayesian Information Criterion (BIC), Figure S4: Results from the comparison of Model 1 and Model 2 based on Akaike’s weights and ΔBIC.
