# Supplementary material for: Attempts at the Characterization of In-Cell Biophysical Processes Non-Invasively—Quantitative NMR Diffusometry of a Model Cellular System
Source: Cells. 2020 Sep 19;9(9):2124. doi: 10.3390/cells9092124 (PMC7565294; doi:10.3390/cells9092124)
Supplement: Supplementary file 1 [file cells-09-02124-s001.pdf]

Supplementary Materials

Attempts at the characterization of in-cell biophysical processes non-invasively- quantitative NMR diffusometry of a model cellular system

Weronika Mazur [1,2,*] and Artur T. Krzyżak [2]

[1] Faculty of Physics and Applied Computer Science, AGH University of Science and Technology, ul. Reymonta 19, 30-059 Cracow, Poland; Weronika.Mazur@fis.agh.edu.pl

[2] Faculty of Geology, Geophysics and Environmental Protection, AGH University of Science and Technology, al. Mickiewicza 30, 30-059 Cracow, Poland; akrzyzak@agh.edu.pl

* Correspondence: Weronika.Mazur@fis.agh.edu.pl

Table of Contents

S1. Materials and methods 2

Figure S1: The construction scheme of the NMR-MoUSE device.. 2

Figure S2: The Stimulated Spin Echo (SSE) pulse sequence used in the experiment. 3

Table. S1: Parameters of the protocol used in diffusion measurements. 3

Figure S3: Log-log plot of diffusion coefficient dependency on diffusion time 4

S2. Theory of model comparison based on Akaike's (AIC) and Bayesian Information Criterion (BIC) 4

Figure S4: Results from the comparison of Model 1 and Model 2 based on Akaike's weights and ΔBIC 6

Bibliography 6

S1. Materials and methods

The construction scheme of the NMR-MoUSE device applied for diffusion measurements is presented in Figure S1.

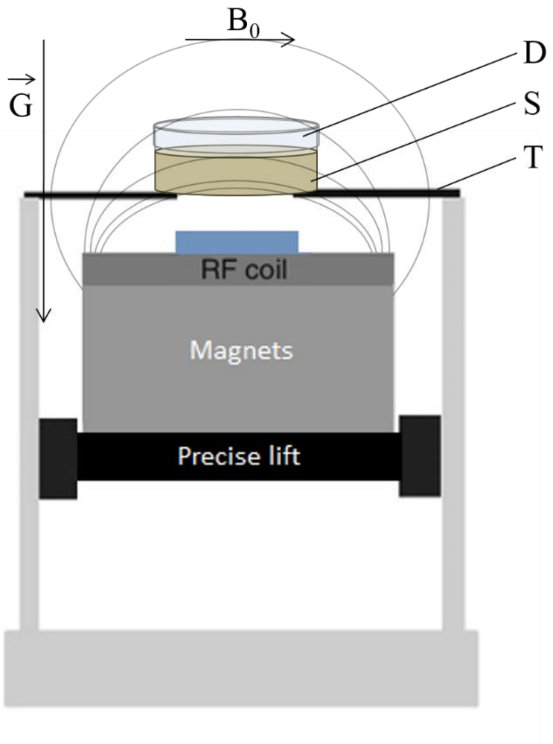

Figure S1: The construction scheme of the NMR-MoUSE device. RF coil and permanent magnets are attached to the high precision lift, the movement of which controls the depth that is excited. B_0 is the magnetic induction of a stray field generated by Magnets; $\vec{G}$is the magnetic field vector. D denotes for Petri Dish (closed) that is placed on table T and contains the sample S.

Diffusion was measured using the SSE pulse sequence (Figure S2). In this sequence, the magnetization vector is rotated three times by the angle of 90° via RF pulses. In that way the directions of magnetic field gradient and magnetization vector are aligned only during concrete time intervals. During these intervals, magnetization "feels" the magnetic field gradient as it was a pulse of duration τ, which is the time between the two 90° pulses. During the time interval t_m magnetization does not "feel" the gradient (it is stored in the direction of a magnetic field) and then the mixing of magnetic moments occurs, i.e. water diffuses. After that time, a third RF pulse is applied which rotates the magnetization vector to the direction of the gradient and, after time τ, the stimulated echo (SSE) is produced.

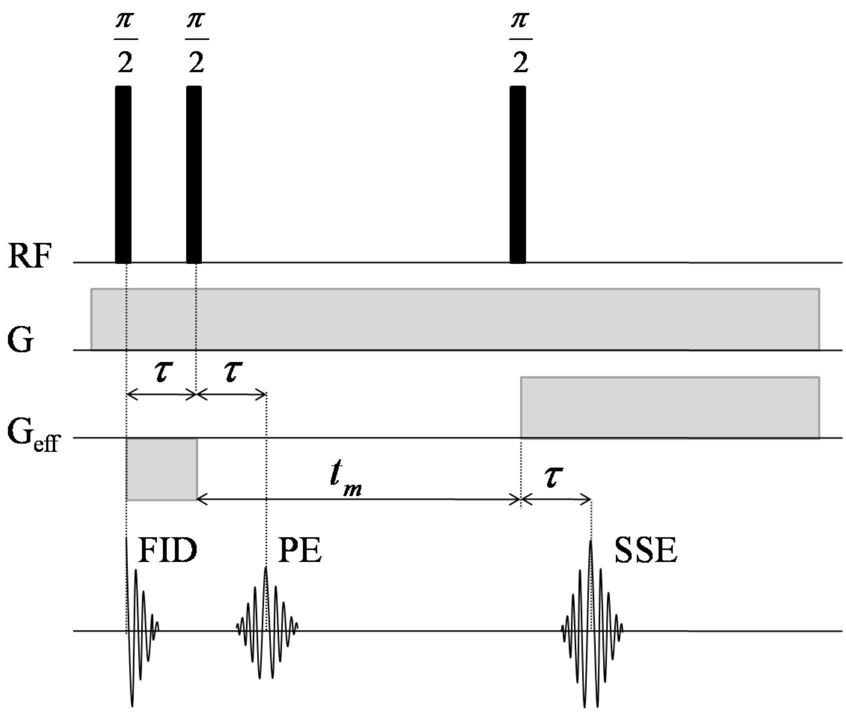

Figure S2: The Stimulated Spin Echo (SSE) pulse sequence used in the experiment. Constant gradient G is present during the whole procedure, but magnetization "feels" the effective gradient G_{eff}. After the first RF pulse magnetization is rotated to the direction of magnetic field gradient and Free Induction Decay (FID) is produced. After the time τ the second RF pulse is applied and magnetization is stored in the B_0 direction for the period of t_m during which Primary Echo (PE) is produced and mixing of magnetic moments occurs. After t_m the third RF pulse is applied which refocuses the magnetic moments and after τ the SSE is produced.

The values of parameters in the SSE sequence used in the experiments are summarized in Table S1.

Table S1. Parameters of the protocol used in diffusion measurements. τ_{min} and τ_{max} are the minimal and maximal diffusion encoding times, respectively; *N* is a number of diffusion encoding time steps in each experiment; t_m is mixing time; *RD* is repetition delay; *NoS* is number of scans; b_{min} and b_{max} are the minimal and maximal diffusion weighting factors, respectively.

t_m (ms)	τ_{min} (ms)	τ_{max} (ms)	*N*	*RD* (ms)	*NoS*	b_{min} (sm^{-2})	b_{max} (sm^{-2})
0.2	0.02	0.6	20	1500	128	0	8.87E+09
0.4						3.29E+06	1.18E+10
0.6						6.57E+06	1.48E+10
0.8						9.86E+06	1.77E+10
1						1.31E+07	2.07E+10

2						2.96E+07	3.55E+10
5						7.89E+07	7.98E+10
7						1.12E+08	1.09E+11
10						1.61E+08	1.54E+11
12						1.94E+08	1.83E+11
14						2.27E+08	2.13E+11
16						2.60E+08	2.42E+11
20						3.25E+08	3.02E+11
24						3.91E+08	3.61E+11
40						6.54E+08	5.97E+11

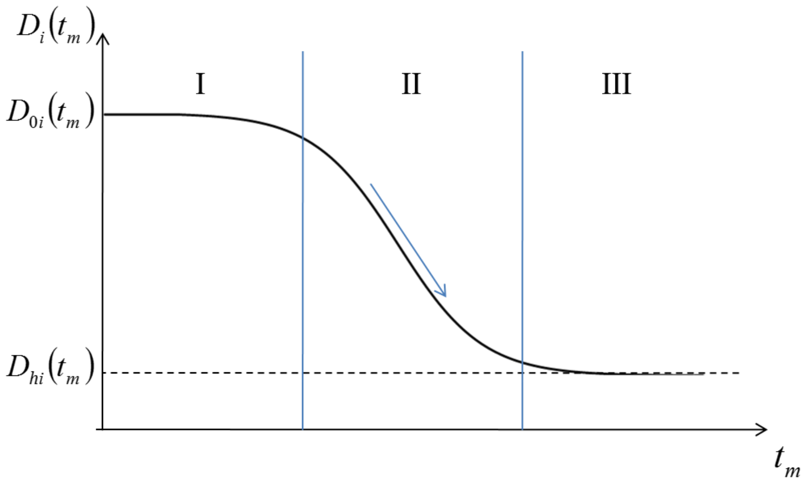

Figure S3. Log-log plot of diffusion coefficient dependency on diffusion time (based on [1]). I- region of a free diffusion regime; II- region of restricted diffusion (localization regime), the beginning of which describes the eq. (3) and the arrow indicates the area where the eq. (4) stands for diffusion behavior; III- region of hindered diffusion (motional averaging regime), where increasing the diffusion time further does not influence the diffusion coefficient D_{hi} (t_m).

S2. Theory of model comparison based on Akaike's (AIC) and Bayesian Information Criterion (BIC)

The appropriate model of diffusion in the samplecan be chosen based on Akaike's Information Criterion (AIC) and Bayesian Information Criterion (BIC), which is a modification of AIC. If i=1, 2,…, k is the number of models, the general relation for AIC_i is given by

$$AIC_i = N_i \cdot ln\left(\frac{RSS_i}{N_i}\right) + 2K_i, \qquad when\,\frac{N}{K} \geq 40 \qquad \text{(S1a.)}$$

$$AIC_i = N_i \cdot ln\left(\frac{RSS_i}{N_i}\right) + 2K_i + \frac{2K_i(K_i+1)}{N_i-K_i-1}, \qquad when\,\frac{N}{K} < 40, \qquad \text{(S1b.)}$$

where N_i is a number of data points, K_i is a number of fitted parameters and RSS_i is a Residual Sum of Squares, i.e. the sum of the squared vertical distances between fitted function and data point, in the *i*-th model. Equation (S1b.) is called corrected AIC for a finite sample and is used in the following analysis. If the two models are compared, those one with the smaller AIC is supposed to be true. BIC_i is given by

$$BIC_i = N_i \cdot ln\left(\frac{RSS_i}{N_i}\right) + K_i \cdot ln(N_i), \qquad \text{(S2)}$$

and from which it can be seen that it has a penalty term larger compared to the penalty term in the AIC when $N > e^2$. Despite the similarity between AIC and BIC, they are calculated based on different frameworks (while BIC assumes that a true model is in the set of analyzed models, AIC does not). Thus, both criteria are used to see what information they will deliver about the models, since they address different questions.

A very convenient way of making inferences about the true model is based on Akaike's weights, which indicates the probability of a better model. If AIC_{min} corresponds to the lowest AIC among models, then $\Delta AIC_i = AIC_i - AIC_{min}$, and

$$Akaike's\ weight = \frac{e^{-0.5\Delta AIC_i}}{1+e^{-0.5\Delta AIC_i}}. \qquad \text{(S3)}$$

In the case of BIC, the interpretation can be based on the ΔBIC, and if the BIC_{min} corresponds to the lowest BIC among models, then

$$\Delta BIC_i = BIC_i - BIC_{min}. \qquad \text{(S4)}$$

The ΔBIC_i value can be interpreted as follows

$$If\ \Delta BIC_i \begin{cases} < 2, & then\ BIC\ is\ inconclusive \\ > 10, & then\ BIC gives\ decisive\ conclusion\ that\ model\ is\ correct \end{cases}. \qquad \text{(S5)}$$

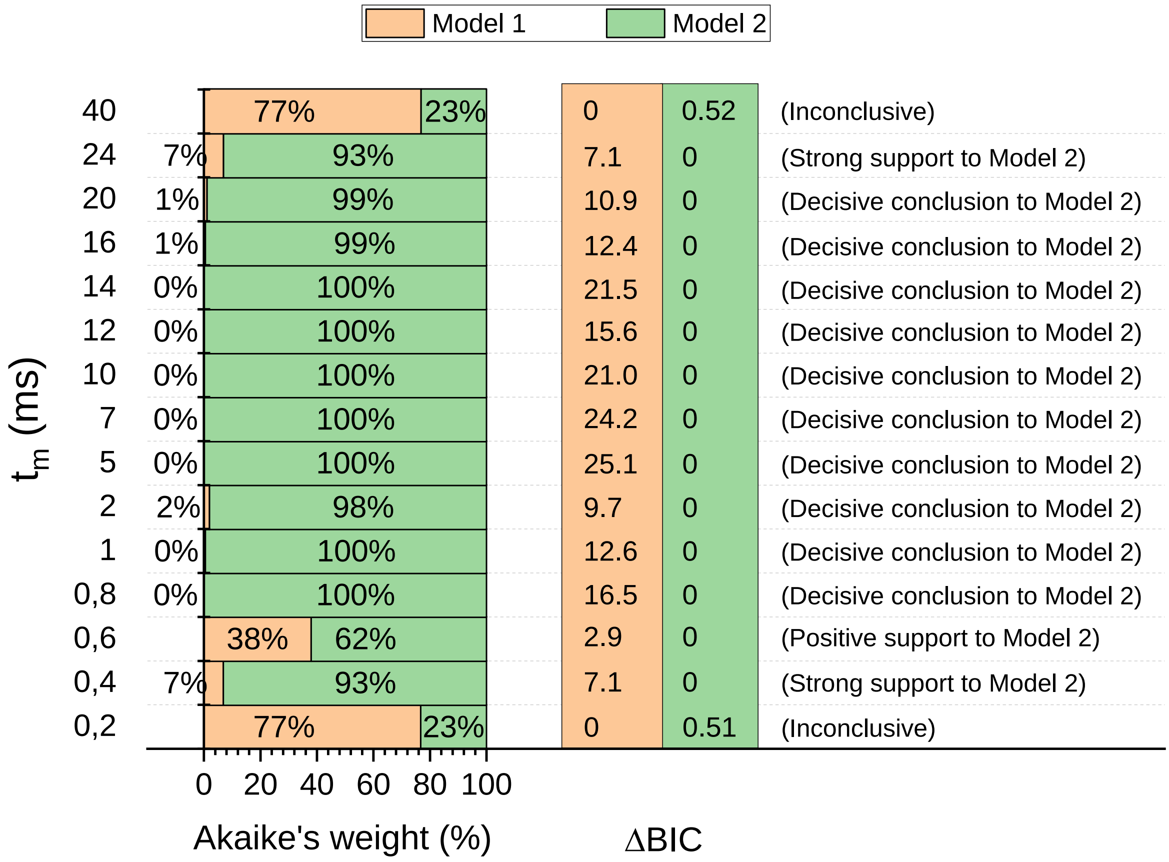

Figure S4: Results from the comparison of model 1 and model 2 based on Akaike's weights and ΔBIC.

References

1. Cho, C.; Hong, Y.; Kang, K.; Volkov, V.I.; Skirda, V.; Lee, C.J.; Lee, C. Water self-diffusion in Chlorella sp. studied by pulse field gradient NMR. *Magn. Reson. Imaging* **2003**, *21*, 1009–1017, doi:10.1016/S0730-725X(03)00206-6.
